# Effect of Relative Humidity on Adsorption Breakthrough of CO_2_ on Activated Carbon Fibers

**DOI:** 10.3390/ma10111296

**Published:** 2017-11-11

**Authors:** Yu-Chun Chiang, Yu-Jen Chen, Cheng-Yen Wu

**Affiliations:** 1 Department of Mechanical Engineering, Yuan Ze University, Taoyuan 32003, Taiwan; s1020922@mail.yzu.edu.tw (Y.-J.C.); s1020947@mail.yzu.edu.tw (C.-Y.W.); 2 Fuel Cell Center, Yuan Ze University, Taoyuan 32003, Taiwan

**Keywords:** relative humidity, carbon dioxide, activated carbon fibers, surface modification, adsorption breakthrough

## Abstract

Microporous activated carbon fibers (ACFs) were developed for CO_2_ capture based on potassium hydroxide (KOH) activation and tetraethylenepentamine (TEPA) amination. The material properties of the modified ACFs were characterized using several techniques. The adsorption breakthrough curves of CO_2_ were measured and the effect of relative humidity in the carrier gas was determined. The KOH activation at high temperature generated additional pore networks and the intercalation of metallic K into the carbon matrix, leading to the production of mesopore and micropore volumes and providing access to the active sites in the micropores. However, this treatment also resulted in the loss of nitrogen functionalities. The TEPA amination has successfully introduced nitrogen functionalities onto the fiber surface, but its long-chain structure blocked parts of the micropores and, thus, made the available surface area and pore volume limited. Introduction of the power of time into the Wheeler equation was required to fit the data well. The relative humidity within the studied range had almost no effects on the breakthrough curves. It was expected that the concentration of CO_2_ was high enough so that the impact on CO_2_ adsorption capacity lessened due to increased relative humidity.

## 1. Introduction

As interests over the impact of the emissions of greenhouse gases on global warming continue to increase, especially the rising concentration of carbon dioxide (CO_2_) primarily from the use of fossil fuels, it is imperative to alleviate CO_2_ emissions by development of pollution prevention technologies. The CO_2_ capture and sequestration (CCS) is a set of technologies that can greatly reduce CO_2_ emissions and is considered an effective approach in mitigating global warming [1,2,3,4]. A typical untreated flue gas composition from a power plant burning low sulfur eastern bituminous coal consists of approximately 15–16% CO_2_, 70–75% N_2_, 5–7% H_2_O, 3–4% O_2_, and several trace amounts of constituents [5]. Various CO_2_ capture technologies, such as absorption, adsorption, cryogenics, and membranes have been widely investigated [4,6]. Although the absorption–regeneration technologies are recognized as the most effective methods and are widely implemented, the high energy density of the absorption processes has restricted their applications. On the other hand, adsorption has been considered as one of the most cost-effective options for CO_2_ separation because of the low energy requirement, cost advantage, and ease of applicability over a relatively wide range of temperatures and pressures [7].

The key point to success of adsorption technology depends on the development of the adsorbents with a high CO_2_ selectivity and adsorption capacity, easy regeneration, and high stability. Several possible adsorbents, including activated carbon fibers (ACFs) [4], activated carbon [8], zeolite [9], silica adsorbents [10], metal organic frameworks (MOFs) [11], or carbon nanotubes (CNTs) [12], have been considered as potential candidates for CO_2_ capture. In addition, surface modification techniques are usually requisite and have been used to improve the CO_2_ adsorption capacity of carbonaceous materials. For instance, the chemical activation of ACFs by potassium hydroxide (KOH) [4] or the functionalization of mesoporous capsules using oligomeric amine [13]. Lee and Park [4] observed that the KOH-modified ACFs had the narrowest microporosity ranging from 0.5 nm to 0.7 nm and exhibited higher CO_2_ adsorption capacity. The KOH-activated nitrogen-containing granular porous carbons with developed microporosity between 0.43 and 1 nm and high N content showed high CO_2_ adsorption capacity at one bar and 25 °C [14]. However, the CO_2_ adsorption capacity was dominated by its surface area and pore volume when the adsorption pressure was higher than five bar. The tetraethylenepentamine (TEPA)-loaded TiO_2_ nanotubes exhibited a better CO_2_ adsorption capacity due to their higher amino-group content and the CO_2_ adsorption capacity was enhanced under varying moisture conditions [15].

In order to estimate carbon bed breakthrough time (service time) for a given gas or vapor which is removed from flowing air by physical adsorption, both the adsorption capacity and adsorption rate need to be known [16]. Several theoretical or empirical equations have been proposed to fit the breakthrough curves in fixed-bed adsorption, such as the Wheeler equation [17,18] or the Wheeler–Jonas equation [19], the modified Wheeler equation [20], the Mecklenburg equation [21], and the Yoon and Nelson equation [22]. The Wheeler equation was originally used to describe the behavior of adsorbable poison gas on catalytic reactors [23] and modified by Jonas and Svirbely [17] to study tetrachloride and chloroform adsorption on activated carbon. Wood [24], however, introduced the skew parameter to quantify the effect of unsymmetrical breakthrough curves. The Wheeler equation can be used successfully to describe any type of adsorption of a single vapor by a suitable adsorbent [19]. The reason that this equation was widely used could be attributed to its apparent simplicity: The combination of a single capacity term and an overall kinetic effect, which strongly enhanced its applicability to different adsorption circumstances. Zhou et al. [25] used the Wheeler equation to discuss xenon breakthrough on activated carbon, and found that the adsorption rate coefficient was proportional to the values of flow rate, but was independent of that of temperature and inlet concentration.

It has been reported that water vapor would be competitively adsorbed on zeolite and blocked the access for CO_2_ [26]. In addition, the MOFs had high capacity of CO_2_ at high pressure, however, their capacities were lower at atmospheric pressures [27]. Although various aminopropylsilanes have been grafted onto mesoporous silicas to produce excellent adsorption capacities of CO_2_ [28], the costs of these aminopropylsilanes and mesoporous silicas (adsorbents) were too high for large-scale applications in CO_2_ capture [29]. In contrast to the poor adsorption capacity of CO_2_ on zeolites in the presence of moisture, the carbon materials were relatively insensitive to moisture [30] and were suitable candidates for CO_2_ capture in flue gas.

Generally, ACFs exhibit a high surface area, large surface density, high mass transfer rates (or smaller mass transfer resistance) for both adsorption and desorption and are easier to handle compared to granular and powdered activated carbons [31]. Cheng et al. [32] proposed a new mathematical model to predict the breakthrough curves of volatile organic compounds on ACFs fixed beds, where both pore diffusion and surface diffusion were considered. They estimated the pore diffusion coefficient was similar to the surface diffusion coefficient. Brasquet and Cloirec [31] observed that the main resistance to the mass transfer for adsorption on ACFs might be due to the diffusion through micropores within the ACFs and the effect of the external mass transfer was insignificant.

The objective of this study was to investigate the breakthrough characteristics of CO_2_ on ACFs under varying moisture conditions. Once the adsorption capacity and adsorption rate were obtained, the service time in ACF application for CO_2_ capture could be estimated. To the best of our knowledge, the studies investigating the effects of relative humidity for CO_2_ adsorption on carbon materials were sparse and their equations of breakthrough curves have not been studied. One commercial ACF cloth was modified by KOH activation or TEPA amination and the materials’ properties were characterized. Their adsorption breakthrough curves for carbon dioxide (CO_2_) were measured and the effect of relative humidity in the gas stream was discussed.

## 2. Experimental Methods

### 2.1. Modification of ACFs

One commercial polyacrylonitrile (PAN)-based ACFs cloth sample was used as the starting material (AW1107, denoted as ACFC), which was provided by Taiwan Carbon Technology Co. (Taichung, Taiwan). In order to increase the micropore volume, the surface of ACFC was activated using KOH (30603, Sigma-Aldrich, Seelze, Germany). The KOH activation process was initiated by mixing ACFC with KOH in deionized water with a weight ratio of KOH to ACF = 2. The mixing was sonicated for 10 min and then dried for 24 h at 100 °C. Next, the mixture was transferred in a ceramic boat into a tubular furnace for thermal treatment. Activation conditions were 800 °C for 1 h at a heating rate of 10 °C/min under flowing N_2_ of 100 sccm. The activated product was washed using hydrochloric acid (HCl, 1 M, Sigma-Aldrich, St. Louis, MO, USA) and deionized water to remove the residual KOH and then dried at 100 °C for 24 h. This sample was denoted as KOH–ACFC.

In addition, the surface of ACFC was immersed with TEPA to decorate the nitrogen functionalities for improving the interactions between CO_2_ molecules and the ACFC. The TEPA amination was conducted as follows. About 1.5 g of ACFC sample was impregnated with 21.8 mL of TEPA solution (10 wt % in alcohol). The mixing was sonicated for 90 min at 60 °C and then dried for 24 h at 50 °C. Next, the mixture was positioned in a ceramic boat into a tubular furnace for thermal treatment at 500 °C for 1 h at a heating rate of 10 °C/min under flowing N_2_ of 100 sccm. The product was washed with deionized water to remove the residual TEPA and then dried at 100 °C for 24 h. This sample was denoted as TEPA–ACFC.

### 2.2. Characterizations of ACFs

The properties of the samples were characterized using several techniques. The surface morphology of the samples was observed using field emission scanning electron microscopy (FESEM, Hitachi S-4800, Hitachi, Krefeld, Germany). The surface structure of the samples were probed by N_2_ adsorption/desorption isotherms measured at −196 °C, carried out using an ASAP 2020 (Micromeritics, Norcross, GA, USA) accelerated surface and porosimetry analysis system. X-ray photoelectron spectroscopy (XPS) was a technique which analyzed the elements constituting the sample surface, its composition, and chemical bonding state by irradiating X-rays on the sample surface, and measuring the kinetic energy of the photoelectrons emitted from the sample surface. The XPS spectra of all samples were obtained using a spectrophotometer (PHI 5000 VersaProbe II, ULVAC-PHI, Kanagawa, Japan), where the scanning X-ray monochromator (Al Anode, h*ν* = 1401 eV) was used and the information on elements within a few nanometers of the sample surface could be obtained. For calibration purposes, the C_1s_ electron binding energy that corresponds to graphitic carbon was set at 284.6 eV. A nonlinear least squares curve-fitting program (XPSPEAK software, version 4.1, The Chinese University of Hong Kong, Hong Kong, China) was used for deconvolution of the XPS spectra. 

### 2.3. Breakthrough Curves of CO_2_

The breakthrough curves of CO_2_ adsorption on all samples were measured at 25 °C and different relative humidity levels (0, 45, 55, 65, or 75%) according to the ASTM D5160-95 method [33]. The adsorption temperature was set at room temperature to make the measurements available for comparison to studies in the literature. In addition, according to the study of Suzuki [34], the adsorption isotherms of water vapor on ACFs at 25 °C showed minimal adsorption at relative humidity values greater than 40%. The adsorption amounts would go up with an abrupt slope at 60–70% relative humidity and then level off. Thus, this study chose five relative humidity levels covering this interval. The main items in the adsorption system were a single packed adsorbent bed and a humidifier system. The adsorption bed was composed of a stainless steel mesh column with an effective working length of 5 cm, and an outside diameter of 1.5 cm. The ACF sample was wound around the outside of the stainless steel mesh column with two circular layers. The column was in a cylinder reactor with an internal diameter of 3 cm and a height of 15 cm. The weight of the adsorbent was about 1.0–1.5 g. A thermocouple was positioned at the adsorption column to monitor the temperature of the adsorption bed. According to the references [5,35], the typical untreated flue gas compositions from a power plant burning low sulfur coal contained about 15–16% of CO_2_. Thus the CO_2_ concentration was set at 15% quantified by gas chromatography with thermal conductivity detector (GC/TCD), which was supplied by the CO_2_ and N_2_ cylinders under appropriate mixing. The moist gas mixture was obtained by bubbling water using N_2_ and the relative humidity was measured by a temperature hygrometer (HC2-S, Rotronic AG, Zurich, Switzerland). The total flow rate of the gas stream was 100 sccm. The CO_2_ concentration at the exit was measured using GC/TCD. The Wheeler equation, Equation (1) [18], was used for data fitting to determine the gravimetric capacities (*W_e_*) and the average overall rate coefficients (*k_v_*). Since the original one could not fit the data well, a modified equation was proposed Equation (2):

(1) $$t=\frac{W_{e}\;W}{C_{o}\;Q}-\frac{W_{e}\;\rho_{b}}{k_{v}\;C_{o}}\ln\left(\frac{C_{o}-C}{C}\right)$$

(2) $$t^{n}=\frac{W_{e}\;W}{C_{o}\;Q}-\frac{W_{e}\;\rho_{b}}{k_{v}\;C_{o}}\ln\left(\frac{C_{o}-C}{C}\right)$$

where *t* was the breakthrough time (min), *W_e_* was the equilibrium adsorption amount (g/g), *W* was the weight of adsorbent (g), *C_o_* was the inlet concentration (g/cm^3^), *C* was the outlet concentration at time *t* (g/cm^3^), *Q* was the flow rate (cm^3^/min), *ρ_b_* was the bulk density of adsorption bed (g/cm^3^), *k_v_* was the overall adsorption rate coefficient (1/min), and *n* was the power of time. Regeneration of the adsorbents saturated with CO_2_ was measured to see if the adsorbents can be reused. After the concentration of CO_2_ effluent achieved the inlet concentration, desorption was carried out counter currently under dry N_2_ flow at 100 sccm by thermal regeneration at 150 °C until there was no CO_2_ detected in the effluent gas. Successive breakthrough experiments were conducted.

## 3. Results and Discussion

**Figure 1:**
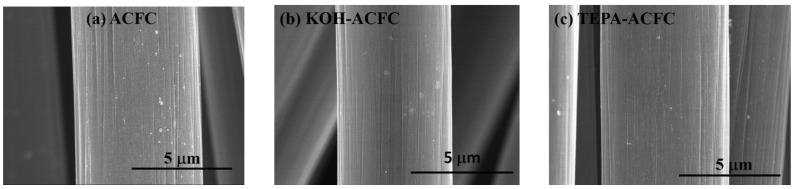
FESEM images of the activated carbon fiber cloth (ACFC) samples: (**a**) as-received ACFC; (**b**) KOH-activated ACFC (KOH–ACFC); and (**c**) TEPA-modified ACFC (TEPA–ACFC).

The FESEM images of the adsorbents are shown in Figure 1. The diameter of a single fiber of ACFC as-received was approximately 6.0 μm and the surface was smooth with typically well-defined striations almost parallel to the fiber axis (Figure 1a). After KOH activation process, there was no significant change in morphology and the diameters of the fibers were narrowed slightly (Figure 1b). This could be due to the etching of the carbon framework by the redox reactions between various K-containing compounds with carbonaceous materials, which was responsible for further generating pore network [36]. The TEPA amination also did not make a visible change in the apparent morphology of the fibers (Figure 1c). TEPA was featured with higher amino-groups content and less viscous nature compared to several amines [13,15].

Table 1 shows the surface characteristics of the adsorbents, which were determined from the N_2_ adsorption/desorption isotherms of the samples at –196 °C. The adsorption isotherms for all samples were essentially type I according to the classification proposed by Brunauer, Emmett, and Teller [37]. Since the c value in Brunauer–Emmett–Teller (BET) surface area report was negative, it implied the BET surface area was invalid. The c value is the BET constant, which is defined as 1 + A/I, where A and I are the slope and the *y*-intercept in a BET plot. Therefore, the Langmuir model was used to find the specific surface areas in this study. The Langmuir specific surface areas of ACFC, KOH–ACFC and TEPA–ACFC were 1385, 2304, and 1051 m^2^/g, respectively. The total pore volume and micropore volume for all samples have the similar trend. Furthermore, the ratios of micropore volume to total pore volume were 0.80 (ACFC), 0.79 (KOH–ACFC), and 0.73 (TEPA–ACFC). As seen from the data, KOH activation of ACFs generated a lot of mesopore and micropore volumes. Moreover, KOH–ACFC exhibited the highest specific surface area, and its microporosity still retained. Table 1 also recorded the volume of micropores with diameter less than 1 nm (fine pores), which followed the order KOH–ACFC > ACFC > TEPA–ACFC. The fact that KOH–ACFC had the largest fine pore volumes could be due to a series of processes [36,38]: The chemical activation resulted in the generation of pore network, the physical activation contributed to the further development of porosity through carbon gasification, and the intercalation of metallic K into the carbon matrix. The metallic K was very active and mobile at the activation temperature so that the lattices of carbon matrix expanded and leading to microporosity. It was expected that the long chain structure of TEPA molecules could block the original micropores and further decrease the available surface area and pore volume. The ratios of fine pore volume to micropore volume were 0.51 (ACFC), 0.55 (KOH–ACFC), and 0.47 (TEPA–ACFC), respectively. It is believed that KOH activation generated a high fraction of fine pores (<1 nm) [39]. The mean equivalent pore widths from Dubinin–Astakhov (DA) method were approximately 1.6 nm, corresponding to the optimum pore width for CO_2_ adsorption in literature [40], though Thiruvenkatachari et al. [41] reported them as 1.8 nm.

The XPS survey scan spectra of the samples revealed that the major peaks in the scan spectra were due to the C_1s_, O_1s_, and N_1s_ photoelectrons. Table 2 summarized the surface atomic contents and atomic ratios. The as-received ACFC contained about 2.43 at.% of N_1s_ because of the PAN precursor and 8.20 at.% of O_1s_. The nitrogen content on KOH–ACFC significantly decreased compared to ACFC, implying the loss of N-moieties during KOH activation at high temperature, which was consistent with the results in several literature [39,42,43,44]. On the other hand, the TEPA amination of ACFC resulted in a large increase in N_1s_ contents, although it suffered a heat treatment at 500 °C. It verified that TEPA has been grafted onto the surface of ACFC.

**Figure 2:**
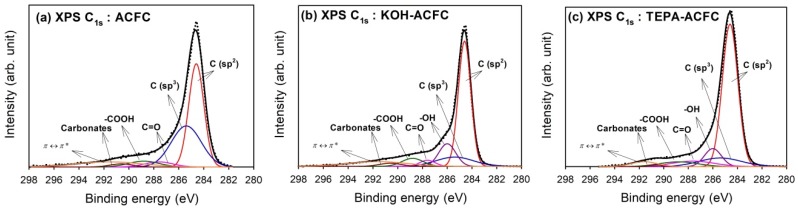
High-resolution fitted XPS C_1s_ spectra of the activated carbon fiber cloth (ACFC) samples: (**a**) as-received ACFC; (**b**) KOH-activated ACFC (KOH–ACFC); and (**c**) TEPA-modified ACFC (TEPA–ACFC).

In order to understand the chemical bonding states, the deconvolution of high-resolution XPS spectra over the C_1s_, O_1s_, and N_1s_ regions for all samples was conducted. Figure 2 illustrates the optimum curve fitting of the high-resolution XPS C_1s_ spectra for all samples and Table 3 shows the calculated percentages of non-functional and functional carbon atoms. We found that the C_1s_ spectra were decomposed into at most seven identified components that represented carbon atoms in polyaromatic structures (C (sp^2^), B.E. = 284.6 eV) and in aliphatic structures (C (sp^3^), B.E. = 285.4 eV), and carbon presented in phenolic, alcohol, ether or C=N groups (B.E. = 286.0 eV), carbonyl or quinone groups (B.E. = 287.6 eV), carboxyl, lactone, or ester groups (B.E. = 288.8 eV), carbonate groups (B.E. = 290.6 eV), and satellite peaks due to π–π* transitions in aromatic rings (B.E. = 291.6 eV) [45]. The C (sp^2^) and C (sp^3^) were predominant in C_1s_ region. An increase in C (sp^2^) and a decrease in C (sp^3^) displayed the improvement of graphite-like structure after modification. The –COOH groups were the major functional groups in ACFC. After KOH activation, the –OH groups increased significantly. On the other hand, the TEPA amination resulted in the increase of –OH groups and C=O, which indicated the grafting of TEPA molecules [46].

**Figure 3:**
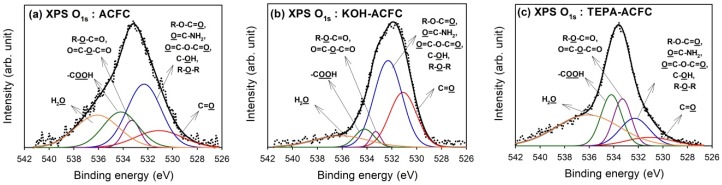
High-resolution fitted XPS O_1s_ spectra of the activated carbon fiber cloth (ACFC) samples: (**a**) as-received ACFC; (**b**) KOH-activated ACFC (KOH–ACFC); and (**c**) TEPA-modified ACFC (TEPA–ACFC).

Deconvolution of XPS O_1s_ peaks gave additional information on the nature of the surface oxygen-containing groups. The optimum curve fitting of the O_1s_ peak for all samples are shown in Figure 3 and the calculated percentages of oxygen-containing functional groups are shown in Table 4. Four different O functionalities and the contribution of chemisorbed water can be identified [45]. The peak at 531.1 eV corresponded to the carbonyl oxygen atoms; the peak at 532.3 eV to the carbonyl oxygen atoms in esters, amides and anhydrides as well as oxygen atoms in hydroxyls or ethers; the peak at 533.3 eV to the ether oxygen atoms in esters and anhydrides; and the peak at 534.2 eV to the oxygen atoms in the carboxyl groups. The contribution of chemisorbed water located at 536.1 eV (H_2_O) was found in all samples. As seen from the data, the oxygen atoms in KOH–ACFC were mainly the –OH and C=O groups. However, the chemisorbed water was the primary surface oxide on TEPA–ACFC.

**Figure 4:**
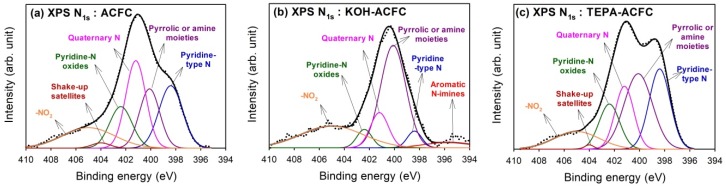
High-resolution fitted XPS N_1s_ spectra of the activated carbon fiber cloth (ACFC) samples: (**a**) as-received ACFC; (**b**) KOH-activated ACFC (KOH–ACFC); and (**c**) TEPA-modified ACFC (TEPA–ACFC).

Figure 4 shows the results of curve-fitting for the high-resolution XPS N_1s_ spectra for all samples, which found that the N_1s_ spectra were decomposed into at most six identified components [47]. The calculated percentages of nitrogen-containing functional groups are shown in Table 5. The peak at 395.7 eV represented the aromatic N-imines; the peak at 398.4 eV was the pyridine-type N; the peak at 400.1 eV showed the pyrrolic or amine moieties; the peak at 401.2 eV was the quarternary N; and the peak at 402.4 eV exhibited the pyridine-N oxides. The contribution of chemisorbed NO_2_ was located at 405 eV. Except for the chemisorbed NO_2_, the quarternary N and the pyridine-type N were the predominant N-functionalities on ACFC, while the grafting of TEPA would lead to the increase in pyrrolic or amine moieties which implied the linear TEPA resulted in the increase of pyridine-like structures of six-member or five-member rings. Although KOH activation resulted in the decrease in N content, the major N groups on KOH–ACFC were the pyrrolic or amine moieties and the chemisorbed NO_2_. It was believed that the pyrrolic or amine moieties were stable compared to the pyridine-type N and quaternary N, and that the KOH activation destroyed the pyridine N structure.

**Figure 5:**
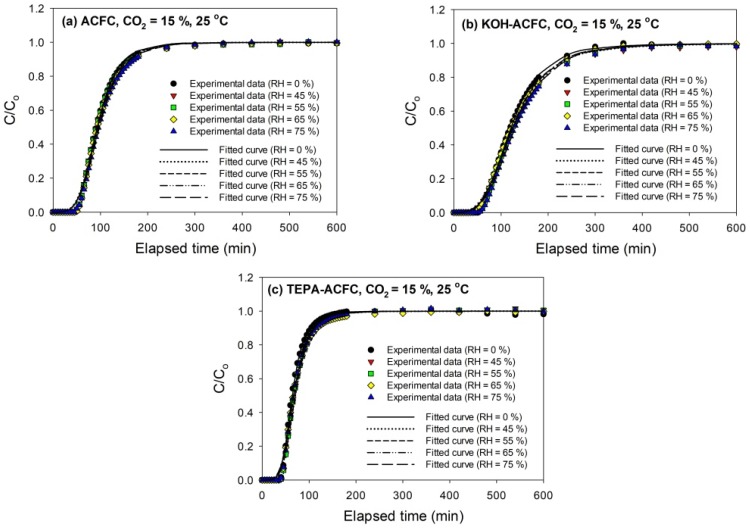
Adsorption breakthrough curves of CO_2_ at 25 °C on activated carbon fiber cloth (ACFC) samples: (**a**) as-received ACFC; (**b**) KOH-activated ACFC (KOH–ACFC); and (**c**) TEPA-modified ACFC (TEPA–ACFC).

The breakthrough curves of CO_2_ (15%) on all adsorbents at 25 °C are sketched in Figure 5. The breakthrough curves obtained with KOH–ACFC were less steep, suggesting a higher pore diffusion resistance than with the other two adsorbents [31], which could be attributed to its large micropore volume. As seen in Figure 5, it implies that the relative humidity within the studied range (45–75%) had almost no effects on the breakthrough curves, which is consistent with the study of Li et al. [48]. It was expected that the interactions between CO_2_ molecules and the ACFs were stronger than those between the water vapor molecules and the ACFs such that the competitive adsorption of water vapor on ACFs was insignificant [49,50]. 

**Figure 6:**
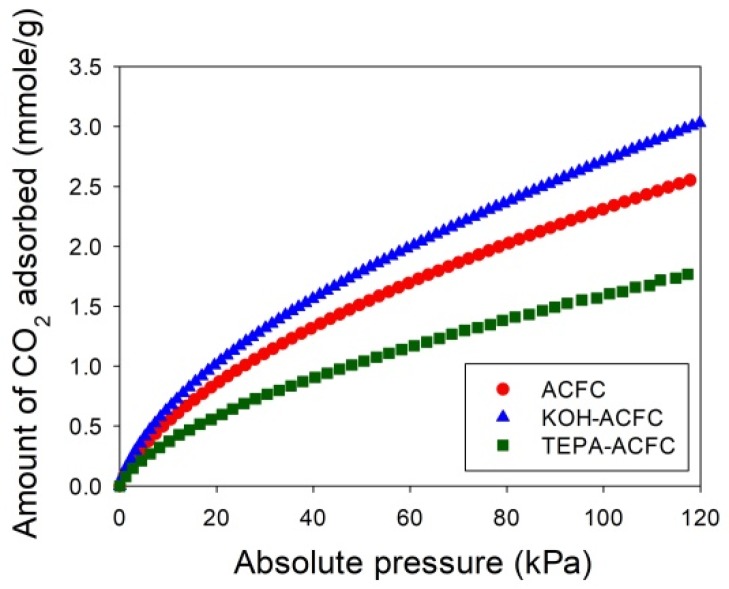
Adsorption isotherms of CO_2_ at 25 °C of the activated carbon fiber cloth (ACFC) samples.

Since the original Wheeler equation (Equation (1)) could not fit the data well around the breakthrough point (i.e., *C*/*Co* = 0.1) and the *W_e_* was highly overestimated, another parameter, *n* (the power of time), was added to the model. To determine an appropriate value of *n*, the adsorption isotherms of CO_2_ were required. Figure 6 depicts the adsorption isotherms of CO_2_ collected on the adsorbents at 25 °C for low CO_2_ pressures less than 120 kPa, measured using an ASAP 2020 (Micromeritics, Norcross, GA, USA). The temperature during the CO_2_ adsorption process was maintained by a circular water bath thermostat. The uptakes of CO_2_ had typical monotonic curves, which were increasing with increasing pressures and indicative of the favorable adsorption. The adsorption amounts of CO_2_ at 25 °C and 15 kPa followed the order KOH–ACFC (0.855 mmole/g) > ACFC (0.713 mmole/g) > TEPA–ACFC (0.486 mmole/g). These equilibrium data were utilized to calibrate the values of *n*. After the curve fitting process, it produced *n* = 0.1 for ACFC, *n* = 0.08 for KOH–ACFC, and *n* = 0.001 for TEPA–ACFC, respectively. The reason that impregnating amine did not result in increase of CO_2_ uptake could be attributed to the low TEPA loading [13]. Therefore, the increase in CO_2_ uptake due to carbamate formation was limited.

The above fitted values of *n* were kept unchanged for each adsorbent. Then the modified Wheeler equation was used further to fit the breakthrough data measured at different relative humidity for each adsorbent. The fitting reports are shown in Table 6. The high values of *k_v_* for TEPA–ACFC were responsible for the steep breakthrough curves. The equilibrium adsorption amounts, *W_e_*, exhibited a little increase with increasing relative humidity on ACFC and KOH–ACFC. However, the values of *k_v_*, the overall adsorption rate coefficient, was decreased in the presence of water vapor. This appeared that the adsorption rate was surface adsorption limited because the active sites were occupied by water vapor molecules or it was pore diffusion limited such that the adsorption rate slowed down and the values of *k_v_* decreased [18]. 

**Figure 7:**
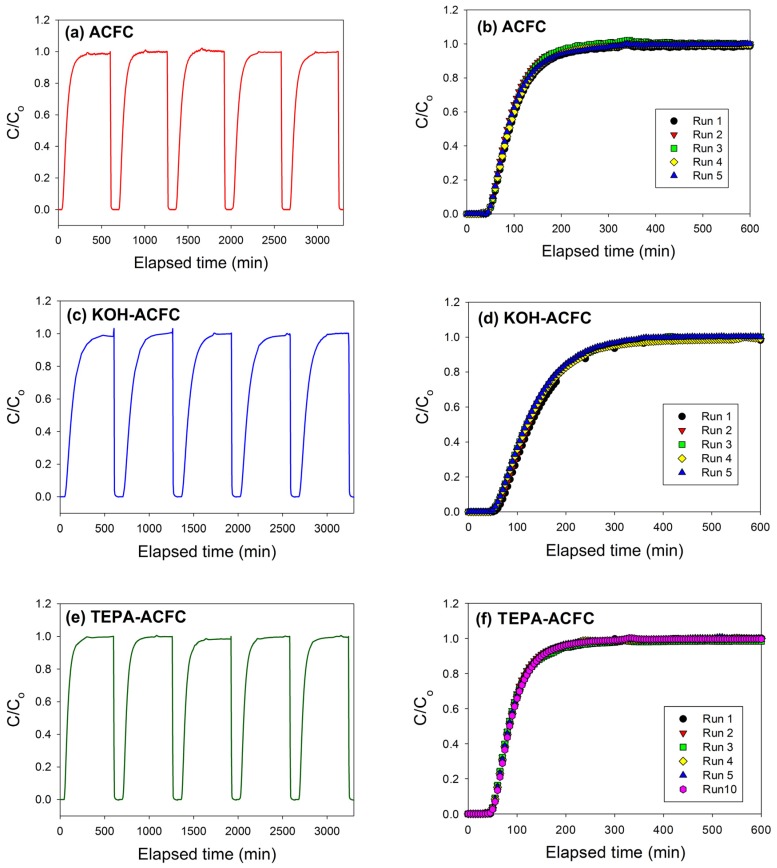
Several successive cyclic adsorption/desorption breakthrough curves: (**a**,**b**) as-received ACFC; (**c**,**d**) KOH–activated ACFC (KOH–ACFC); and (**e**,**f**) TEPA-modified ACFC (TEPA–ACFC).

**Figure 8:**
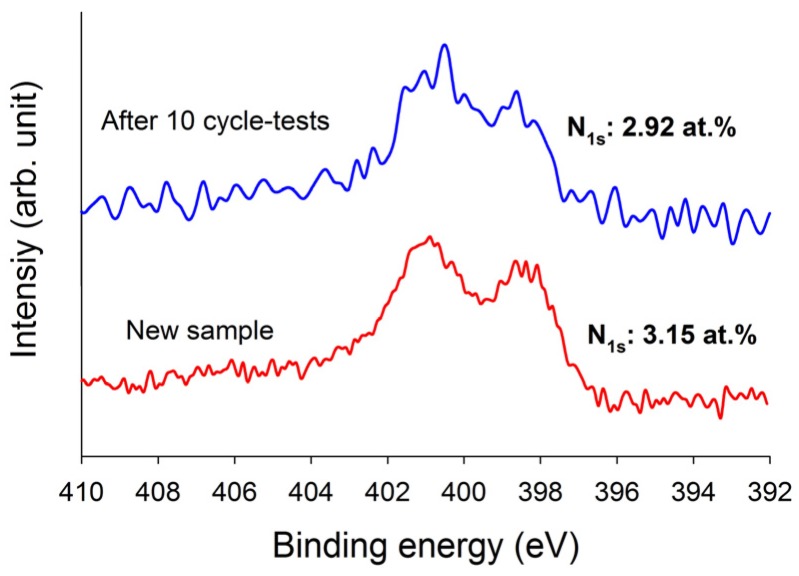
The nitrogen contents for the new TEPA–ACFC sample and the samples after 10 adsorption/desorption cycle-tests (by thermal regeneration) using XPS.

In order to check the recyclability of the adsorbents, cycle tests were performed for these three adsorbents. As shown in Figure 7, it is evident that these three adsorbents can be used up to five adsorption/desorption cycles almost without any changes in breakthrough time and dynamic adsorption capacities. These revealed that all adsorbents were fairly stable in five adsorption/desorption cycles and the heel (the residual adsorbate present in the bed following regeneration) was insignificant. In addition, the stability of the N-functionalities on TEPA–ACFC during thermal regeneration was analyzed. The nitrogen contents for the new TEPA–ACFC sample and the samples after 10 cycle-tests (by thermal regeneration) were probed using XPS. Their breakthrough curves and XPS N1s spectra are compared in Figure 7f and Figure 8, respectively. It can be observed that two patterns are similar. And the loss of nitrogen atoms on TEPA–ACFC after ten adsorption/thermal regenerations processes was estimated about 7%. It is believed that the N-functionalities were grafted and fixed strongly on ACFC. Table 7 compares the CO_2_ dynamic adsorption capacities on carbonaceous adsorbents. The KOH–ACFC showed a higher CO_2_ uptake compared to those published data in literature under similar operation conditions.

## 4. Conclusions 

Results showed that the morphology of the activated carbon fiber cloth samples almost kept intact after modification. The KOH activation at high temperature resulted in smaller diameter of fibers, the loss of nitrogen functionalities, and the increase in surface area and pore volume. The TEPA amination was an effective way to introduce nitrogen onto the surface of fibers, which has been verified using XPS analysis. However, its long chain structure could block the original micropores and further to decrease the available active sites and pore volume. The modified Wheeler equation with a new parameter, the power of time, fitted the data better than the original one, dependent on the nature of the adsorbent. The micropores controlled the steepness of the breakthrough curves, implying the mass transfer was pore diffusion limited. The equilibrium adsorption capacity and adsorption rate of carbon dioxide at a concentration of 15% on the adsorbents were not influenced by the presence of moisture. Although the pore width remains an important factor in determining CO_2_ adsorption, the pore volume and surface area should be taken into account for evaluation. This study proposed the equations of breakthrough curves in ACF application for CO_2_ capture and the service time could be estimated after the operation conditions are given. Further studies conducted at higher relative humidity levels or at higher adsorption temperatures will be the scope of future experimental work.

## Figures and Tables

**Table 1:** Surface characteristics of the samples determined from N_2_ adsorption/desorption isotherms at –196 °C.

Sample	Langmuir Surface Area (m^2^/g)	Micropore Area ^α^ (m^2^/g)	Total Pore Volume ^β^ (cm^3^/g)	Micropore Volume ^γ^ (cm^3^/g)	Mesopore Volume ^η^ (cm^3^/g)	Macropore Volume ^ϕ^ (cm^3^/g)	Micropore Volume (<1 nm) ^ξ^ (cm^3^/g)	Mean Equivalent Pore width ^ζ^ (nm)
ACFC	1385	957	0.4854	0.3862	0.0669	0.0323	0.1960	1.615
KOH–ACFC	2304	1546	0.7937	0.6261	0.1414	0.0262	0.3439	1.620
TEPA–ACFC	1051	657	0.3678	0.2672	0.0527	0.0479	0.1247	1.628

^α^ Micropore area was determined by Dubinin–Astakhov (DA) method. ^β^ Total pore volume (V_t_) represents the single point total pore volume at P/P_o_ ≅ 0.99. ^γ^ Micropore volume (V_mi_) was determined by DA method. ^η^ Mesopore volume (V_me_) was found by Barrett–Joyner–Halenda (BJH) method. ^ϕ^ Macropore volume (V_ma_) was found by subtracting V_mi_ and V_me_ from V_t_. ^ξ^ Micropore volume (<1 nm) was determined by non-local density functional theory (NLDFT) method. ^ζ^ Mean equivalent pore width was determined by DA method.

**Table 2:** Surface atomic ratios of the samples from XPS analysis.

Sample	Atomic Ratio (%)			O/C	N/C
	C_1s_	N_1s_	O_1s_		
ACFC	89.37	2.43	8.20	0.092	0.027
KOH–ACFC	89.26	0.69	10.05	0.113	0.008
TEPA–ACFC	91.02	3.15	5.83	0.064	0.035

**Table 3:** Results of the fits of the XPS C_1s_ region, values given in at.% of total intensity.

Sample	Binding Energy (eV)						
	284.6	285.4	286.0	287.6	288.8	290.6	291.6
	C (sp^2^)	C (sp^3^)	–OH	C=O	–COOH	Carbonates	π-π*
ACFC	44.3	34.9	-	4.0	5.1	2.0	9.7
KOH–ACFC	53.8	11.9	13.2	3.2	5.8	2.4	9.7
TEPA–ACFC	64.1	10.6	8.9	6.7	5.4	0.6	3.7

**Table 4:** Results of the fits of the XPS O_1s_ region, values given in at.% of total intensity.

Sample	Binding Energy (eV)				
	531.1	532.3	533.3	534.2	536.1
	C=O	R–O–C=O, O=C-NH_2_, O=C–O–C=O, C–OH, R–O–R	R–O–C=O, O=C–O–C=O	–COOH	H_2_O
ACFC	13.1	35.5	7.5	20.5	23.5
KOH–ACFC	29.3	48.1	3.2	7.0	12.4
TEPA–ACFC	7.6	16.0	16.7	20.9	38.9

**Table 5:** Results of the fits of the XPS N_1s_ region, values given in at.% of total intensity.

Sample	Binding Energy (eV)						
	395.7	398.4	400.1	401.2	402.4	404	405
	Aromatic N-imines	Pyridine-type N	Pyrrolic or Amine Moieties	Quaternary N	Pyridine-N Oxides	Shake-up Satellites	NO_2_
ACFC	—	22.6	18.4	26.9	14.3	1.5	16.4
KOH–ACFC	4.9	4.5	48.2	11.8	5.2	—	25.5
TEPA–ACFC	—	24.9	31.4	17.4	13.8	0.6	11.9

**Table 6:** Results of the fits of the CO_2_ adsorption breakthrough curve using the modified Wheeler equation (CO_2_: 15%, temperature: 25 °C).

Adsorbent	Relative Humidity (%)	*k_v_* (1/min)	*W_e_* (g/g)	*n*	*R^2^*	Breakthrough Time * (min)	*W_e_^#^* (g/g)
ACFC	0	770	0.030170	0.1	0.99736	60.8	0.031357
	45	751	0.030167	0.1	0.99771	59.8	-
	55	753	0.030193	0.1	0.99768	60.4	-
	65	744	0.030254	0.1	0.99726	61.5	-
	75	738	0.030312	0.1	0.99829	61.8	-
KOH–ACFC	0	818	0.03668	0.08	0.99841	70.2	0.037633
	45	740	0.036761	0.08	0.99790	68.5	-
	55	780	0.036784	0.08	0.99841	70.8	-
	65	768	0.036741	0.08	0.99860	68.8	-
	75	787	0.036887	0.08	0.99795	73.8	-
TEPA–ACFC	0	90521	0.022067	0.001	0.99721	45.6	0.021365
	45	84368	0.022069	0.001	0.99765	47.3	-
	55	83808	0.022069	0.001	0.99752	47.2	-
	65	79088	0.022068	0.001	0.99670	46.2	-
	75	81729	0.022068	0.001	0.99766	46.3	-

* The breakthrough point was set at *C*/*C_0_* = 0.1. ^#^ The equilibrium adsorption amount of CO_2_ was measured at 15 kPa and 25 °C.

**Table 7:** Comparisons of the CO_2_ dynamic adsorption capacities on carbonaceous adsorbents.

Adsorbent	Modification Chemicals	Conditions *	*W_e_* (mg/g)	Reference
Activated carbon fibers	-	C_o_: 15%, T: 25 °C, RH: 0–75%	30	This work
Activated carbon fibers	KOH	C_o_: 15%, T: 25 °C, RH: 0–75%	37	This work
Activated carbon fibers	TEPA	C_o_: 15%, T: 25 °C, RH: 0–75%	22	This work
Norit R1 Extra	-	C_o_: 15%, T: 25 °C, RH: 0%	24	Dreisbach et al. [51]
Silica-coated multi-walled carbon nanotubes	Polyethylene -imine	C_o_: 15%, T: 25 °C, RH: 0%	29	Lee and Park [52]
Multi-walled carbon nanotubes	3-aminopropyl-triethoxysilane	C_o_: 15%, T: 20 °C, RH: 0%	43	Su et al. [12]
Mesoporous alumina	-	C_o_: 15%, T: 55 °C, RH: 10%	13	Thote et al. [53]
Mesoporous alumina	-	C_o_: 15%, T: 55 °C, RH: 0%	29	Thote et al. [53]
Activated carbon fiber	-	C_o_: 100%, T: 25 °C, RH: 0%	31	Moon and Shim [54]
Activated carbon fiber	-	C_o_: 15%, T: 25 °C, RH: 0%	35~70	Lee and Park [4]

* C_o_: the inlet concentration of CO_2_, T: adsorption temperature, and RH: relative humidity.
